# Rapid Biodegradation of the Organophosphorus Insecticide Chlorpyrifos by *Cupriavidus nantongensis* X1^T^

**DOI:** 10.3390/ijerph16234593

**Published:** 2019-11-20

**Authors:** Taozhong Shi, Liancheng Fang, Han Qin, Yifei Chen, Xiangwei Wu, Rimao Hua

**Affiliations:** Key Laboratory for Agri-Food Safety, School of Resource & Environment, Anhui Agricultural University, Hefei 230036, Anhui, China; tzs@ahau.edu.cn (T.S.); fang8232335@ahau.edu.cn (L.F.); HanQin@ahau.edu.cn (H.Q.); ahaucyf2016@ahau.edu.cn (Y.C.); wxwahau@ahau.edu.cn (X.W.)

**Keywords:** biodegradation, chlorpyrifos, *Cupriavidus nantongensis* X1^T^, OpdB

## Abstract

Chlorpyrifos was one of the most widely used organophosphorus insecticides and the neurotoxicity and genotoxicity of chlorpyrifos to mammals, aquatic organisms and other non-target organisms have caused much public concern. *Cupriavidus nantongensis* X1^T^, a type of strain of the genus *Cupriavidus*, is capable of efficiently degrading 200 mg/L of chlorpyrifos within 48 h. This is ~100 fold faster than *Enterobacter* B-14, a well-studied chlorpyrifos-degrading bacterial strain. Strain X1^T^ can tolerate high concentrations (500 mg/L) of chlorpyrifos over a wide range of temperatures (30–42 °C) and pH values (5–9). RT-qPCR analysis showed that the organophosphorus hydrolase (OpdB) in strain X1^T^ was an inducible enzyme, and the crude enzyme isolated in vitro could still maintain 75% degradation activity. Strain X1^T^ can simultaneously degrade chlorpyrifos and its main hydrolysate 3,5,6-trichloro-2-pyridinol. TCP could be further metabolized through stepwise oxidative dechlorination and further opening of the benzene ring to be completely degraded by the tricarboxylic acid cycle. The results provide a potential means for the remediation of chlorpyrifos- contaminated soil and water.

## 1. Introduction

Chlorpyrifos (*O*,*O*-diethyl-*O*-(3,5,6-trichloro-2-pyridinol) phosphorothioate, is an organophosphorus insecticide, nematicide, and acaricide, which was first commercialized in the USA by Dow Chemical Co. (Midland, MI, USA) in 1965 [1]. Chlorpyrifos has been widely used to protect a wide variety of crops, including cereals, fruits, vines, vegetables, ornamentals, cotton and other economic crops to control rice leaf moth, plant hoppers, gall midge, wheat army worm, cotton boll worm, aphid and red spider [2]. Chlorpyrifos also had an effect against soil insects and livestock parasites. However this widespread and frequent application of chlorpyrifos has caused several toxicological, environmental contamination and residue problems, which seriously threaten human health, as well as ecological and environment security [3,4]. To date, residues have been detected in marine sediments, rivers, lakes, groundwater, urban sewage systems, and even in rain and air in the United States and Mexico [5]. Along the Mediterranean coast, especially in Turkey, chlorpyrifos residues were the most high-frequency insecticide detected in environment [6].

Various toxic effects caused by chlorpyrifos have been reported. Previous studies have shown that chlorpyrifos not only has acute and chronic toxicity to mammals, aquatic organisms and other non-target organisms, but also has neurotoxicity, genotoxicity and other multiple toxic influences [7,8]. Therefore, it is urgent to repair any environment contaminated by chlorpyrifos. Numerous significant studies related to the repair of soil and water pollution by chlorpyrifos were reported, including photochemical degradation using physical and chemical methods, nanometal materials or UV/H_2_O_2_ catalytic degradation [9,10]. Compared with these methods, biodegradation is an efficient and environmental friendly method to repair soil and water contaminated by chlorpyrifos [11].

At present, a variety of microorganisms capable of degrading chlorpyrifos have been isolated and identified from the environment, like *Pseudomonas* sp. CB2 [12], *Paracoccus* sp. TRP [13], *Sphingomonas sp.* Dsp-2 [14], *Alcaligenes faecalis* DSP3, *Streptomyces* sp.AC5, *Bacillus cereus* MCAS02, *Cupriavidus* sp. DT-1 [15], *Ochrobactrum* sp. JAS2 [16], etc. 3,5,6-Trichloro-2-pyridinol (TCP) and diethylthiophosphoric acid (DETP) are the main metabolites of chlorpyrifos obtained by biodegradation [17]. TCP has been listed as a persistent and mobile organic pollutant by the U.S. Environmental Protection Agency (EPA) due to its higher solubility in water and longer half-life (65–360 days) than its parent compound chlorpyrifos [18]. It has reported that a combination of chlorpyrifos and TCP could cause greater toxic effects than either alone [19]. However, most bacteria can only degrade chlorpyrifos but not TCP. Only a few bacteria capable simultaneously degrade both chlorpyrifos and TCP [20]. Therefore, it is of great significance to select a strain that can efficient degrade chlorpyrifos and TCP simultaneously.

Chlorpyrifos commonly undergoes three metabolic pathways: (1) Alkylation pathway, where the main metabolite TCP can further generate 3,5,6-trichloro-2-methypyridine (TMP) or 3,5,6-trichloro-2-methoxypyridine (TCMP) by alkylation reactions [21]. (2) Reductive dechlorination pathway, in which TCP is first dechlorinated to chlorodihydro-2-pyridone, and then dechlorinated further to tetrahydro-2-pyridone, and then the pyridine ring is cleaved to form maleamide semialdehyde, and finally mineralized to CO_2_, ammonium ion, water, and other inorganic materials [1]. (3) Oxidative dechlorination pathway. Bhuimbar et al. reported that *Micrococcus luteus* and *Bacillus subtilis* can further dechlorinate the *para* and *ortho*-Cl atoms in TCP to form 3,6-dihydroxypyridyl-2,5-diketone through oxidation reactions [22]. However, some key intermediate metabolites were not detected due to their unstable structures and low concentration.

Microbial degradation of organic pollutants is generally considered to be caused by enzymes. Phosphoric triester hydrolases (EC 3.1.8), the most studied chlorpyrifos-degrading enzymes, include methyl parathion hydrolase (MPH), OP acid anhydrolase (OPAA), OP degradation enzyme (OPD), phosphotriesterase (PTE) and glycerophosphodiesterase (GPD) [23,24]. All these enzymes belong to the metalloenzymes class and have a bivalent cationic bimetallic activity center. Among these enzymes, the substrates of OPD are the most extensive and toxic [25].

*Cupriavidus nantongensis* X1^T^ was recently isolated from an active sludge found at an organophosphorus insecticide manufacturing site in Nantong (Jiangsu Province, China). It was identified to be a novel species (type strain) of the genus *Cupriavidus* and complete genome sequencing showed that strain X1^T^ have the degradation gene opdB (encoding organophosphate hydrolase) in an original plasmid pX1 [26,27]. In this study, the high efficiency ability to degrade chlorpyrifos by strain X1^T^ was evaluated. Optimum degradation conditions (pH, temperature and inoculation) were determined and the degradation kinetics were characterized. A complete metabolic pathway was found and the oxidative dechlorination pathway of chlorpyrifos was improved.

## 2. Materials and Methods

### 2.1. Chemicals and Culture Medium

Chlorpyrifos (purity = 98.5%) was purchased from Dr. Ehrenstorfer GmbH (Augsburg, Germany). The metabolite 3,5,6-trichloro-2-pyridinol (TCP, purity = 95%) was purchased from J&K Scientific (Beijing, China). Chromatographic grade solvents, including methanol, acetonitrile and formic acid, were purchased from Merck (Darmstadt, Germany). All other reagents were analytical or HPLC grade.

The Luria-Bertani (LB) medium contained 10 g/L tryptone, 5 g/L yeast extract powder and 10 g/L NaCl in deionized water at pH 7.0. LB solid medium was prepared by adding 2% agar powder. Mineral salt medium (MSM) contained 1 g/L NaCl, 0.1 g/L MgSO_4_·7H_2_O, 0.3 g/L KH_2_PO_4_, and 1 g/L K_2_HPO_4_ in deionized water at pH 7.0.

### 2.2. Inoculum Preparation

A single colony of activated X1^T^ bacteria was transferred into 100 mL LB culture medium and incubated overnight (about 16 h, at the exponential growth phase) at 37 °C with 150 rpm on a rotary shaker. The cells were harvested after centrifugation (6000 rpm, 10 min) and washed twice with MSM. Then the cells were re-suspended in MSM and adjusted optical density (OD) value to 0.6 (~1 × 10^8^ colony forming unit, CFU) at a wavelength of 600 nm (OD_600_) by UV-1800 type spectrophotometer (Shimadzu Corp., Kyoto, Japan).

### 2.3. Degradation of Chlorpyrifos by Strain X1^T^

Chlorpyrifos was added to MSM as the only carbon source. A standard reaction sample (20 mL) was contained 50 mg/L chlorpyrifos, 18 mL MSM and 2 mL suspensions of strain X1^T^ (OD_600_ = 0.6). All samples were incubated at 150 rpm and 37 °C with three replicates. The negative control was inoculated without cell suspensions of strain X1^T^ under the same conditions. 20 mL pure acetonitrile was added to terminate the reaction and extract the residual chlorpyrifos and metabolites. All tubes were periodically sampled to measure residual chlorpyrifos and metabolites by UPLC.

To achieve optimum conditions for chlorpyrifos degradation by strain X1^T^, the inoculation volumes, pH and temperature were explored separately. The effects of inoculation volumes on the biodegradation of chlorpyrifos was determined at level of 1%, 2%, 5%, 10% and 20% (the total volume of the reaction samples) under the condition of 37 °C and pH 7. The effects of temperature on chlorpyrifos were evaluated at 20, 30, 37, 42 and 47 °C under the condition of 10% inoculation volumes and pH 7. The effects of pH were adjusted at 5, 6, 7, 8 and 9 under the condition of 10% inoculation volumes and 37 °C.

### 2.4. Determination of Chlorpyrifos and TCP

Acetonitrile (20 mL) was added to each sample which was then centrifuged at 6000× *g* for 5 min. The supernatant was filtered through a 0.22-μm PTFE membrane prior to detection of the residues on an Acquity UPLC system (Waters Corp., Milford, MA, USA) coupled with a PDA detector (Waters Corp.) at 300 nm wavelength [20]. Chlorpyrifos and TCP were simultaneously isolated and detected by a BEH C_18_ column (1.7 μm, 2.1 mm × 50 mm) (Waters Corp., Wexford, Ireland). The column temperature was 40 °C. The mobile phase was composed of solvent A (0.1% formic acid in water), and solvent B (0.1% formic acid in acetonitrile) at a constant flow rate of 0.5 mL/min. The gradient elution was programmed to increase the amount of solvent B from an initial 5% (maintained for 0.25 min) to 95% in 2.75 min, stabilize at 95% for 0.5 min, and then return to the initial conditions (5% B) in 0.01 min. The retention time of TCP and chlorpyrifos were 1.937 and 2.919 min, respectively.

### 2.5. RT-qPCR Analysis of the Expression of Chlorpyrifos Degradation Gene

Through previous complete genome sequencing of strain X1^T^, the gene responsible for degradation of chlorpyrifos was identified to be the opdB gene, encoding organophosphorus hydrolase. The relative expression of degradation gene (opdB) was determined by the RT-qPCR method. Strain X1^T^ (10% inoculation) was added to the MSM containing 50 mg/L chlorpyrifos and incubated at 150 rpm and 37 °C. The negative control was inoculated without cell suspensions of strain X1^T^ under the same condition. All tubes were periodically sampled at 0 h, 2 h, 4 h, 8 h and 12 h. The cells were collected and total RNA was extracted by an EasyPure RNA kit (TransGen Biotech, Beijing, China). Then, total RNA was purified by an AccuRT Genomic DNA removal kit (Applied Biological Materials Inc. Vancouver, Canada) and transcripted to cDNA by a TranScript cDNA synthesis supermix kit (TransGen Biotech, Beijing, China). The housekeeping gene (rpoB) was commonly used and selected to compare the expression of chlorpyrifos degradation gene (opdB), previously reported [20]. The primers used were as follows: rpoB-F CTGCTTGCCACCCAGATTGA, rpoB-R AGCCCGTTATGCGAGGAGAT. opdB-F CTAAACGGGCAACGACAGATT and opdB-R CAATGGCGAATTGGGTGTGTA. The RT-qPCR procedure were as follows: polymerase was activated at 95 °C for 3 min, followed by 40 cycles at 95 °C for 10 s and 58 °C for 30 s.

The CT values of reference genes (rpoB) and target gene (opdB) in all samples were determined respectively. The relative expression of the target gene (opdB) to the reference gene (rpoB) was calculated by the following formula:

(1) $$\mathrm{Related}\;\mathrm{expression}\;\mathrm{fold}\;\mathrm{change}=2^{-\Delta \Delta C_{T}}=2^{-\left(C_{t\;\mathrm{opdB}}-C_{t\;\mathrm{rpoB}}\right)-\left(C_{0\;\mathrm{opdB}}-C_{0\;\mathrm{rpoB}}\right)}$$

C_t opdB_ and C_t rpoB_ are C_T_ values of target gene and reference gene at different time, respectively.C_0 opdB_ and C_0 rpoB_ are C_T_ values of target gene and reference gene at initial time, respectively. The related expression of opdB with house-keeping gene rpoB was collected and calculated using the BioRad CFX manager 3.1 software (Hercules, CA, USA).

### 2.6. Determination of the Metabolites of Chlorpyrifos by Strain X1^T^

The metabolites were analyzed on an Acquity UPLC system coupled with a triple quadrupole mass spectrometer (Xevo TQ-MS, Waters Corp. Ltd.) and fitted with an ESI interface and controlled by the MassLynx 4.1 software (Waters, Milford, MA, USA). Metabolites in samples were separated by a BEH C_18_ chromatographic column (1.7 μm, 2.1 mm × 100 mm) and the temperature of column was 30 °C. The mobile phase was composed of solvent A (0.1% formic acid in water) and B (0.1% formic acid in acetonitrile) at a constant flow rate of 0.2 mL/min. The gradient was programmed to increase the amount of solvent B from an initial 5% (maintained for 0.25 min) to 20% in 2 min, stabilize at 20% for 8 min, and then to 95% in 15 min (maintained for 1.0 min) then return to the initial conditions (5% B) in 1.0 min. The mass spectrometer was operated at electrospray ionization mode (ESI) and performed MS1 scan, the scan ranged from *m*/*z* 50 to 400. Positive and negative polarity modes were used simultaneously during the same analytical run. Desolation (400 °C) and ion source (150 °C) were kept at constant temperatures. The voltage was 1000 V and 20 V of capillary and cone, respectively. According to the results of MS1 scan, high responses of molecular ions were found out, and then performed MS2 scan. Scanning range was 50–200 *m*/*z*. Argon (purity > 99.999%) was used as the collision gas at a constant flow of 0.16 mL/min. The metabolites were presumed by the molecular ion and the characteristic fragment ion peaks of the MS2.

### 2.7. Degradation of Chlorpyrifos by Crude Enzyme

Strain X1^T^ (OD_600_ = 0.6, 1 mL) was added to 1 L LB medium and incubated at 150 rpm and 37 °C for 12 h. The cells were harvested by centrifugation (6000× *g* for 10 min) at 4 °C and washed twice with phosphate buffer (50 mM, pH 7.0). The washed cells were resuspended in 25 mL phosphate buffer and disrupted by ultrasonic homogenizer at 250 W by working for 5 s and stopping for 5 s, 100 times. The crude enzyme was extracted by centrifugation at 12,000× *g* for 10 min. The concentration of crude enzyme was detected by Nanodrop One (BioRad, Hercules, CA, USA). The activity of crude enzyme was measured by detecting the degradation of chlorpyrifos. A standard tube was contained 50 mg/L chlorpyrifos, 1 mg/L crude enzyme in phosphate buffer and incubated at constant temperature (37 °C). The negative control was without enzyme under the same condition. The tubes were periodically sampled and the residual of chlorpyrifos was detected by the UPLC method.

### 2.8. Calculation and Statistical Analysis

The data calculation and statistical analysis were performed by Origin 8.0 software (Origin Lab Corp., Northampton, MA, USA) and SPSS 20.0 software (IBM, Armonk, NY, USA). The degradation rate was calculated from the following equation:

(2) $$\mathrm{Degradation}\;\mathrm{rate}\;(\%)=\frac{C_{0}-C_{t}}{C_{0}}\times 100\%$$

The degradation curve was fitted with the first order kinetic equation $\ln\frac{C_{t}}{C_{0}}$ = *k t*. The degradation t_1/2_ was t_1/2_ = ln2/k, where *C*_0_, *C_t_*, *k* are the initial concentration, treatment residual concentration and rate constant, respectively.

## 3. Results

### 3.1. Effects of Inoculation Volume on Chlorpyrifos Degradation by Strain X1^T^

**Figure 1:**
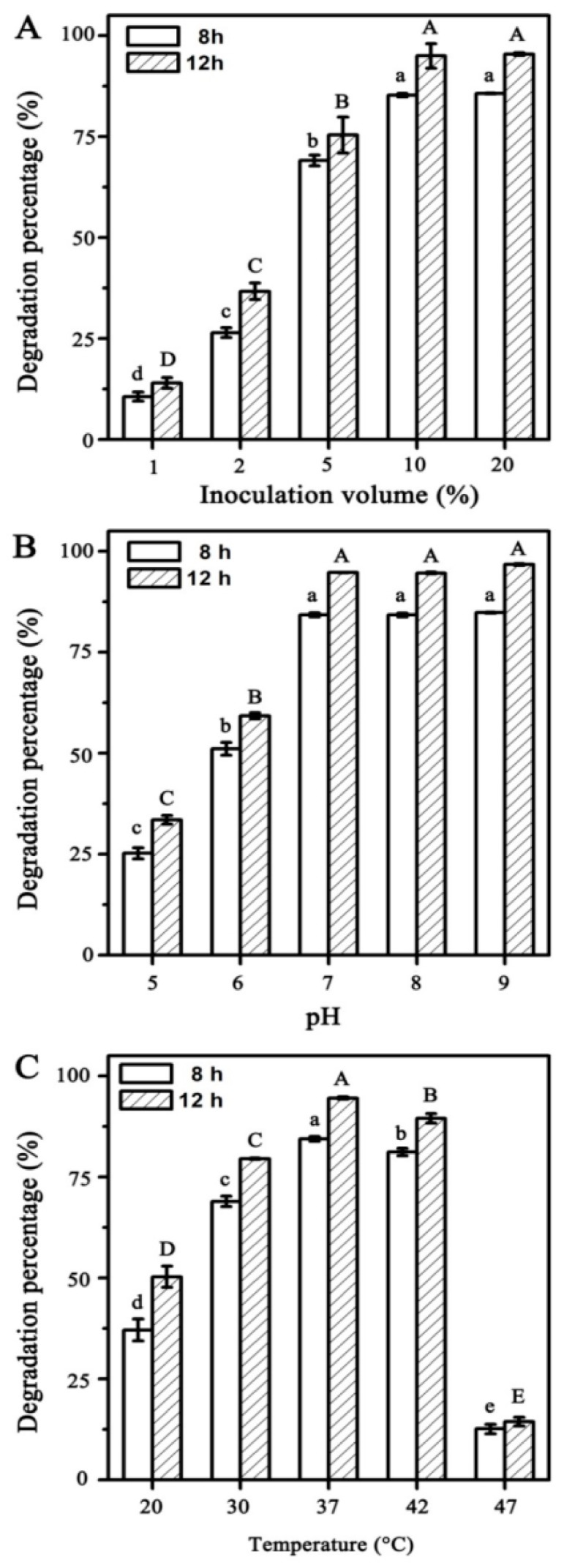
Effects of inoculation volume (**A**), temperature (**B**) and pH (**C**) on chlorpyrifos degradation by strain X1^T^ in 8 and 12 h. (**A**) The effects of inoculation volumes on the biodegradation of chlorpyrifos at level of 1–20% under the condition of 37 °C and pH 7. (**B**) The effects of temperature on chlorpyrifos degradation at 20–47 °C under the condition of 10% inoculation volumes and pH 7. (**C**) The effects of pH on the biodegradation of chlorpyrifos at 5–9 under the condition of 10% inoculation volumes and 37 °C. All values are means ± standard deviation of triplicate measurements. Mean values with the same letter (a, b, c, d, e or A, B, C, D, E) are not significantly different with reaction time (8 or 12 h) by LSD at the 5% level.

As shown in Figure 1A. After 8 h incubation, the degradation rates of 50 mg/L chlorpyrifos were 10.6%, 26.4%, 69.1% and 85.2% with the cell suspension volumes of strain X1^T^ range from 1%, 2%, 5% and 10%, respectively. After 12 h incubation, the degradation rates were increased to 14%, 36.7%, 74.5% and 95%. Under the condition of 10% inoculation volume of strain X1^T^, 50 mg/L chlorpyrifos was nearly complete degraded within 12 h. However, when the inoculation volume was increased to 20%, the degradation rate was not continues increased. The degradation percentage of chlorpyrifos showed no significant difference (*p* < 0.05) under the condition of 10% or 20% inoculation volume after 8 and 12 h. It was shown that the strain X1^T^ had reached saturation under the condition of 10% inoculation volume and further increased the inoculation volume could not improve the degradation efficiency. This indicated that the optimum inoculation volume was 10%.

### 3.2. Effects of pH on Chlorpyrifos Degradation by Strain X1^T^

The degradation of 20 mg/L chlorpyrifos under different pH (5, 6, 7, 8 and 9) by strain X1^T^ were shown in Figure 1B. In the pH range from 5 to 9, strain X1^T^ always shows good degradation activity and the degradation rates were 33.5 ± 1.07, 59.2 ± 0.73, 94.7 ± 0.06, 94.6 ± 0.21 and 96.7 ± 0.19, respectively, after 12 h incubation. These results suggest that chlorpyrifos is more difficult to degrade under acidic conditions. Meanwhile, there was no significant difference (*p* < 0.05) in the degradation effect of chlorpyrifos under alkaline conditions (pH 7 to 9). These results indicated that the optimum pH range for chlorpyrifos degradation by X1^T^ was 5–9.

### 3.3. Effects of Temperature on Chlorpyrifos Degradation by Strain X1^T^

The degradation of 20 mg/L chlorpyrifos by strain X1^T^ at different temperatures (20–47 °C) is shown in Figure 1C. The degradation rates of chlorpyrifos were 50.3 ± 2.64, 79.5 ± 0.18, 94.5 ± 0.32, 89.5 ± 1.14 and 14.4 ± 1.07, respectively, after 12 h incubation in MSM and the degradation activity of strain X1^T^ increased with the increase of temperature (20–42 °C). However, it significantly reduced to 14.4% at 47 °C. These results suggested that the optimum temperature for chlorpyrifos degradation was 30–42 °C.

### 3.4. Degradation Kinetics of Chlorpyrifos by Strain X1^T^

**Figure 2:**
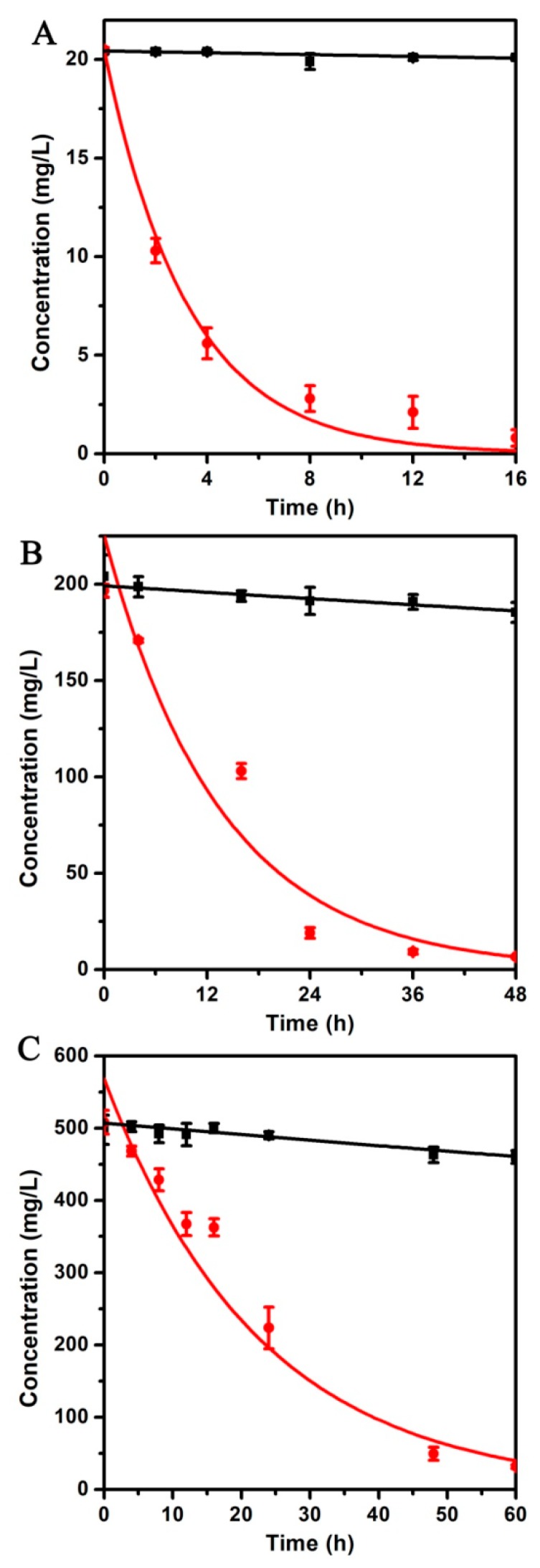
Degradation kinetics of chlorpyrifos with different initial concentration by strain X1^T^. (**A**) 20 mg/L, (**B**) 200 mg/L, (**C**) 500 mg/L. The black line is the negative control (chlorpyrifos without strain X1^T^) and the red line is the positive control (chlorpyrifos with strain X1^T^). All values are means ± standard deviation of triplicate measurements.

Optimum conditions (10% inoculation volume, pH 7 and 37 °C) were applied to detect the degradation of chlorpyrifos at different initial concentrations (20, 200 and 500 mg/L). When the initial concentrations were 20, 200 and 500 mg/L, the degradation rates of chlorpyrifos were 100%, 100% and 90% within 48 h (Figure 2). The degradation curves were well fitted with first-order kinetics and the coefficient R^2^ were 0.9612, 0.9003 and 0.9795, respectively (Table 1). From the degradation rate constant (K), it can be seen that the activity of strain X1^T^ gradually decreases with the increase of the initial concentration of chlorpyrifos. The reason was that the main metabolite of chlorpyrifos, TCP, was toxic to the bacteria. When the degradation rate of TCP was lower than the production rate, TCP would gradually accumulate in the medium and affect the degradation of chlorpyrifos. The minimum inhibitory concentration (MIC) of chlorpyrifos and TCP to strain X1^T^ were also detected by MSM gradient plate. The results showed that the MIC of TCP to strain X1^T^ was 22.3 ± 1.0 mg/L. However, 500 mg/L chlorpyrifos gradient plate cannot detect its bactericidal activity against strain X1^T^. These results further indicate that the decrease of degradation kinetics constants was caused by the metabolite TCP. When the concentration of TCP exceeded the MIC, the degradation activity of strain X1^T^ would be inhibited.

### 3.5. Epression of opdB by RT-qPCR

**Figure 3:**
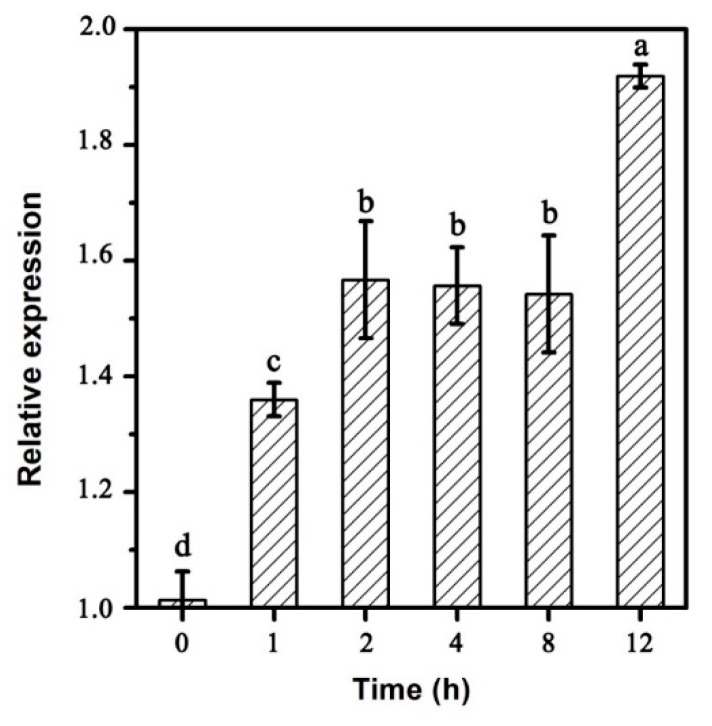
Relative normalized expression of degradation gene (opdB) in degradation process. All values are means ± standard deviation of triplicate measurements. Mean values with the same letter (a, b, c and d) are not significantly different among reaction time (0 to 12 h) by LSD at the 5% level.

The expression of degradation gene (opdB) in chlorpyrifos degradation process was determined by the relative quantitation method with reference gene (rpoB) (Figure 3). Compared with the expression at 0 h, the expression of opdB gradually increased and reached maximum at 12 h as the reaction progressed to indicate that chlorpyrifos could induce the expression of degradation enzyme.

### 3.6. Metabolites and Degradation Pathways of Chlorpyrifos by Strain X1^T^

**Figure 4:**
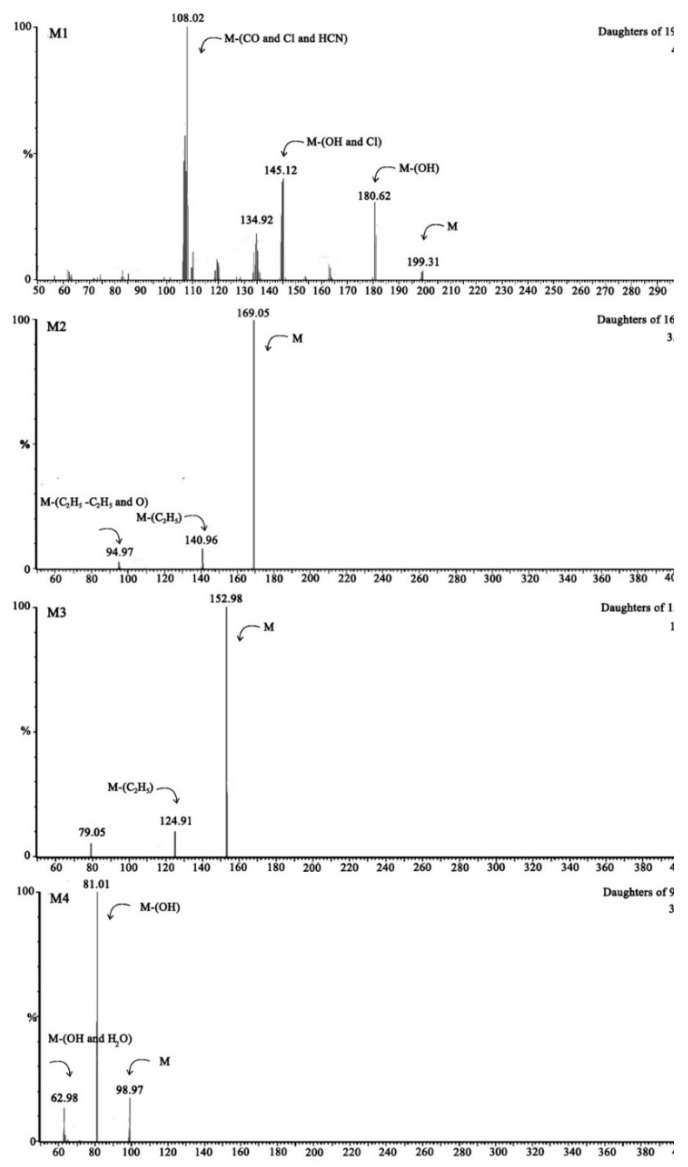

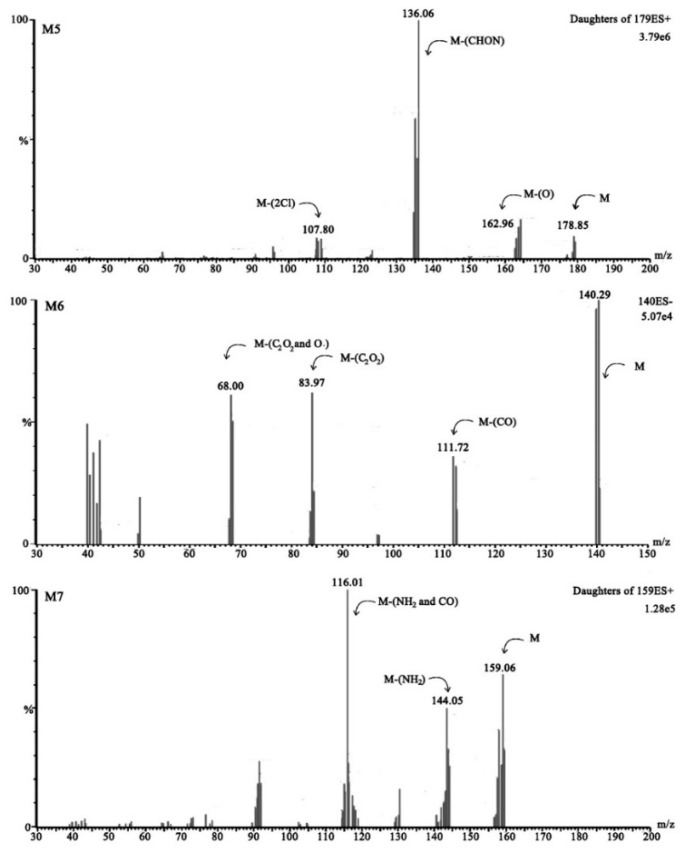
LC-MS/MS analysis of metabolites in the degradation process. (M1) TCP, (M2) DETP, (M3) DEP, (M4) H3PO4, (M5) DCPD, (M6) DHPD, (M7) ATOPA.

The metabolic products of chlorpyrifos were measured and analyzed by UPLC-MS/MS. Seven metabolites (M1 to M7, Figure 4, Table 2) were detected. 

**Figure 5:**
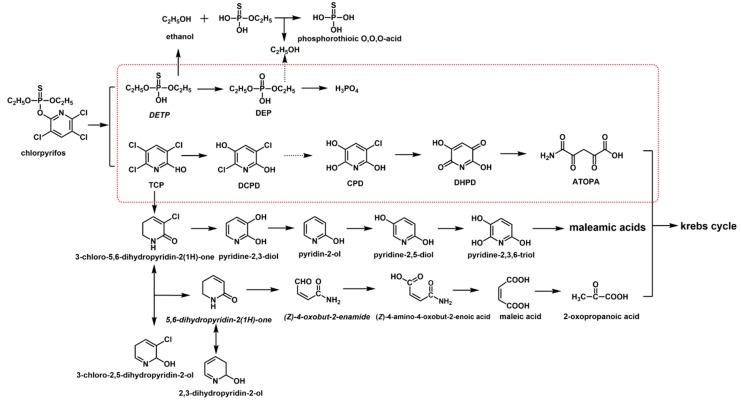
Degradation pathways of chlorpyrifos in strain X1^T^ and other bacteria. The degradation pathway of strain X1^T^ is shown in the red frame.

The prominent protonated [M + 1]^+^ molecular ion of M1 was observed at *m/z* 199.31, and it was identified as TCP by comarison with a standard. The second metabolite of M2 was identified as diethylthiophosphoric acid (DETP) which showed a molecular ion at *m/z* 169.05 [M - 1]^−^, and fragment ions at 140.95 [M - 29]^−^ and 94.97 [M - 74]^−^, indicating losses of H, C_2_H_5_ and C_2_H_5_ + C_2_H_5_ +O. As previously reported M1 and M2 are the main metabolites of chlorpyrifos in hydrolysis processes. Meanwhile, M1 and M2 could be further metabolized into M3, M4, M5, M6 and M7 in strain X1^T^. The degradation product of M3 was identified as diethylacidphosphate (DEP) with a molecular ion at *m/z* 152.98 [M - 1]^−^ and a *m/z* 124.91 [M - 29]^−^, peak, indicating losses of H and C_2_H_5_. The metabolite of M3 could be further metabolized into M4 which was identified as H_3_PO_4_. For TCP degradation, the product M5, with a molecular ion at *m/z* 162.96 [M - 16]^+^, and peaks at 136.06 [M - 43]^+^ and 107.80 [M - 71]^+^ indicating losses of O, CHON and 2Cl was identified as 3,6-dichloro-2,5-dihydroxypyridine (DCPD). M5 could be further dechlorinated by strain X1^T^ to produce M6, which was identified as 3,6-dihydroxypyridine-2,5-dione (DHPD) and showed a molecular ion at *m/z* 111.72 [M-28]^−^, and peaks at *m*/z 83.97 [M-56]^−^ and 68.00 [M-72]^−^, indicating losses of CO, C_2_O_2_, C_2_O_2_ and O. The discovery of M5 and M6 indicated that the degradation process of TCP in strain X1^T^ was a stepwise dechlorination process and all three chlorine atoms on TCP could be removed. The ring opening product of 5-amino-2,4,5-trioxopentanoic acid (M7, ATOPA) with a molecular ion at *m/z* 159.06 [M-1]^+^, and peaks at 143.64 [M-16]^+^, 116.01 [M-44]^+^, indicating losses of H, NH_2_ and CO2, was also detected in the samples. According to the KEGG database, M7 could be converted to maleyl acetate and β-ketoadipate in bacteria, which could enter the tricarboxylic acid cycle and eventually be completely metabolized into carbon dioxide and water. The degradation pathway of chlorpyrifos in strain X1^T^ is shown in Figure 5.

### 3.7. Degradation Activity of Crude Enzyme

**Figure 6:**
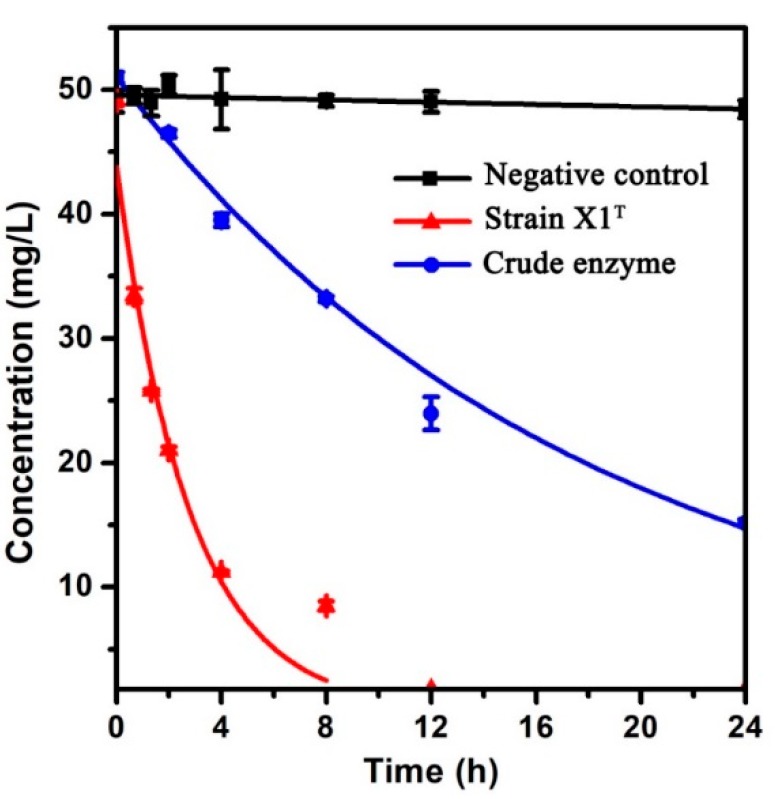
Degradation of 50 mg/L chlorpyrifos by strain X1T and crude enzyme.

To further verify the degradation process, the crude enzyme was extracted. As shown in Figure 6, the crude enzyme extracted from strain X1^T^ could also degrade 50 mg/L chlorpyrifos. However, the degradation ability of crude enzyme was not all as good as (~75%) strain X1^T^ for the same bacterial quantity (OD_600_ = 0.6).

## 4. Discussion

In this study, a high efficient chlorpyrifos-degrading strain, *Cupriavidus nantongensis* X1^T^, was reported. It can use chlorpyrifos as a sole carbon source. A variety of microorganisms capable of degrading chlorpyrifos have been isolated from the environment and identified (Table 3). However, only a few strains (*Paracoccus* sp. TRP [13], *Cladosporium cladosporioides* Hu-01 [28], *Sphingomonas* sp. DSP2 [14] and *Bacillus pumilus* C2A1 [29]) can both biodegrade chlorpyrifos and TCP, but with limited degradation rates. They can take 48–360 h to completely degrade 50–100 mg/L chlorpyrifos and TCP [20,30]. Compared with these degradation strains, strain X1^T^ can degrade chlorpyrifos and TCP simultaneously with high degradation activity.

The degradation rates of chlorpyrifos by strain X1^T^ increased in the temperature range from 20 to 37 °C. The degradation activity remained 90% at 42 °C, but decreased significantly at 47 °C. The results indicate that strain X1^T^ can maintain an efficient degradation activity in a high-temperature environment (37–42 °C). This result is similar to the optimum growth temperature of strain X1^T^. Meanwhile, the degradation ability of the strain is mainly the function of the degradation enzymes in vivo, and the change of temperature will significantly affect the activity of the degradation enzymes [39]. Compared with the optimum temperatures of other organophosphorus hydrolases MPH, PTE and GPD, strain X1^T^ has a higher utilization value in actual chlorpyrifos-contaminated soil remediation [40,41,42].

*Enterobacter* sp. B-14 was reported to rapidly degrade chlorpyrifos under alkaline conditions, while the degradation rate of chlorpyrifos was notably reduced under acidic conditions, which was very similar to the results of strain X1^T^ [43]. One of the main reasons is that organophosphorus insecticides are unstable and more easily hydrolyzed under alkaline conditions [44]. However, different from other degrading strains, strain X1^T^ not only maintains a high degradation activity, but also can tolerate an alkaline environment (the amount of bacteria remains the same).

Different substrate concentrations could also affect the degradation capacity of strain X1^T^. The kinetics results showed that the degradation kinetics constant (k) decreased with the increase of substrate concentration (20–500 mg/L). The degradation kinetics constant at initial concentration of 20 mg/L was 7-fold higher than that of 500 mg/L. One major reason is that TCP, the main metabolite of chlorpyrifos, is toxic and can inhibit the growth of bacteria [1].

Rt-qPCR results showed that the addition of chlorpyrifos could promote the expression of organophosphorus hydrolase (opdB) and with the gradual progress of chlorpyrifos degradation, the expression level gradually decreased. This indicates that organophosphorus hydrolase in strain X1^T^ is an inducible enzyme. Many organophosphorus degradation enzymes have been expressed and the structures were analyzed [45]. All these enzymes isolated so far were belonged to metal enzymes and have a bivalent cationic bimetallic activity center [25]. Different degrading enzymes have optimum substrates. Paraoxonase (with Ca^2+^ as metal ions activity center) displays a preference for sarin and soman [46], OP acid anhydrolase (with Mn^2+^ as metal ions activity center) displays a preference for tabun [47], phosphotriesterase (with Zn^2+^ as metal ions activity center) displays a preference for ethyl-substituted OPs and OP-degrading hydrolases (with Co^2+^ or Cd^2+^ as metal ions activity center) displays a preference for methyl-substituted OPs [25,41]. In present study, OpdB in strain X1^T^ belongs to OP-degrading hydrolases, which could degrade chlorpyrifos and had high degradation activity against methyl parathion, parathion, profenofos, methidathion and isocarbophos [20].

The proposed degradation pathway of chlorpyrifos by strain X1^T^ is shown in Figure 5. Chlorpyrifos was first hydrolyzed to DETP and TCP by OpdB. Then, DETP was oxidized to DEP, which was further degraded to ethanol and phosphoric acid. Meanwhile, TCP could be further degraded to 3,6-dihydroxy-2,5-diketone through stepwise oxidative dechlorination, and then the pyridine ring cleaved to form 5-amino-2,4,5-tricarbonylpentanoic acid, which finally mineralized to CO_2_ and H_2_O by the tricarboxylic acid cycle. This pathway is completely different from the reductive dechlorination pathway also shown in Figure 5. This oxidative dechlorination is similar to that previously reported for 2,4,6-trichlorophenol monooxygenase. However, due to the unstable structure and low content of the dechlorination products, the complete dechlorination process has not been determined. In this study, the complete dechlorination products (with one -Cl, two -Cl and three -Cl removed) were simultaneously detected. Meanwhile, the tcpA gene (encoding 2,4,6-trichlorophenol monooxygenase) was found in chromosome 1 of strain X1^T^ by the previous complete genome sequencing [17]. The optimum degradation substrate and degradation mechanism of 2,4,6-trichlorophenol monooxygenase needs to be further studied after expression and purification.

Chlorpyrifos is one of the pesticides with the highest residue detection rate in the environment. Less than 1% of chlorpyrifos is applied to the target organisms, and most of the rest ends up contaminating the atmosphere, soil and water. Microbial degradation is an efficient and inexpensive way to repair soil and water contaminated by chlorpyrifos. Most of the degrading strains were isolated from activated sludges and pesticide-contaminated soils and they use pesticides as their sole source of carbon and energy for growth. After the decontamination is completed, it is difficult for the degrading strains to continue to grow in the soil and further affect the indigenous microorganisms. In terms of safety issues, the virulence genes were analyzed in the Virulence Factors Database (VFDB) with complete genome sequence of strain X1^T^. According to the similarity threshold of 80%, no virulence genes were found in strain X1^T^. Meanwhile, to further reduce the risk and effects in environment, it is a common method to take the degradation genes into non-pathogenic *E. coli* engineering bacteria and immobilized them in environmentally friendly materials.

## 5. Conclusions

A high efficient chlorpyrifos-degrading strain, *Cupriavidus nantongensis* X1^T^, was isolated and characterized. Strain X1^T^ can tolerate high concentrations of chlorpyrifos under a wide range of temperatures and pH values. The metabolism of chlorpyrifos in strain X1^T^ was a stepwise oxidative dechlorination and the metabolites can further ring-open to reduce the toxicity. The crude enzyme extracted from the strain X1^T^ still maintained good degradation activity. This finding provides a novel valuable material for the remediation in chlorpyrifos and other organophosphorus insecticide- contaminated soil and water.

## Figures and Tables

**Table 1:** Kinetic parameters for degradation of chlorpyrifos by *C. nantongensis* X1^T^.

Compound	Concentration (mg/L)	Degradation Kinetics Equation	k	Half-Life T_1/2_ (h)
Chlorpyrifos	20	C_t_ = 20.49e^−0.308t^ R^2^ = 0.9612	0.308	2.25
	200	C_t_ = 238.02e^−0.071t^ R^2^ = 0.9003	0.071	9.76
	500	C_t_ = 568.20e^−0.044t^ R^2^ = 0.9795	0.044	15.75

**Table 2:** Analysis of metabolites of chlorpyrifos in strain X1^T^ by LC-MS/MS.

Metabolites	Compounds and Molecular Weight (amu)		Molecular Ions (*m*/*z*)	Major Fragments (*m*/*z*)
M1	TCP	198.43	199.31	181[M-OH], 145[M-Cl-OH], 108[M-Cl-CO-HCN]
M2	DETP	170.17	169.05	141[M-C_2_H_5_], 95[M-C_2_H_5_-C_2_H_5_-O]
M3	DEP	154.10	152.98	125[M-C_2_H_5_]
M4	H_3_PO_4_	98.00	98.97	81[M-OH],63[M-OH-H_2_O]
M5	DCPD	180.00	178.85	163[M-O],136[M-CHON],108[M-2Cl]
M6	DHPD	141.08	140.29	112[M-CO], 84[M-C_2_O_2_], 68[M-C_2_O_2_-O]
M7	ATOPA	159.10	159.06	144[M-NH_2_],116[M-NH_2_-CO],

**Table 3:** Degradation rates of strain X1^T^ and other chlorpyrifos-degrading microorganisms.

Strains	Initial Concentration (mg·L^−1^)	Time (day)	Degradation (%)	References
*C. nantongensis*.X1^T^	20	0.67	100	This study
*C. nantongensis*.X1^T^	200	2	100	This study
*C. nantongensis*.X1^T^	500	2	90	This study
*Synechocystis* sp. PUPCCC 64	5	5	93.8	[31]
*Flavobacterium* sp.TCC27551	10	2	100	[32]
*Enterobacter* B-14	20	3	100	[28]
*C. cladosporioides* Hu-01	50	5	100	[33]
*B. cereus* MCAS02	50	7	89	[34]
*Streptomyces* sp. *AC5*	50	1	90	[35]
*Paracoccus* sp. TRP	50	4	100	[36]
*A. faecalis*DSP3	100	12	100	[28]
*Sphingobacterium* sp. JAS3	300	5	100	[37]
*Alcaligenes* sp. JAS1	300	3	100	[38]
